# Barrett's Esophagus: Emerging Knowledge and Management Strategies

**DOI:** 10.1155/2012/814146

**Published:** 2012-05-30

**Authors:** Atul Bhardwaj, Douglas B. Stairs, Haresh Mani, Thomas J. McGarrity

**Affiliations:** ^1^Division of Gastroenterology and Hepatology, Penn State Milton S. Hershey Medical Center, 500 University Drive, P.O. Box 850, HU33, Hershey, PA 17033, USA; ^2^Department of Pathology, Penn State Milton S. Hershey Medical Center, 500 University Drive, P.O. Box 850, HO83, Hershey, PA 17033, USA

## Abstract

The incidence of esophageal adenocarcinoma (EAC) has increased exponentially in the last 3 decades. Barrett's esophagus (BE) is the only known precursor of EAC. Patients with BE have a greater than 40 folds higher risk of EAC compared with the general population. Recent years have witnessed a revolution in the clinical and molecular research related to BE. However, several aspects of this condition remain controversial. Data regarding the true prevalence of BE have varied widely. Recent studies have suggested a lower incidence of EAC in nondysplastic BE (NDBE) than previously reported. There is paucity of prospective data showing a survival benefit of screening or surveillance for BE. Furthermore, the ever-increasing emphasis on healthcare cost containment has called for reexamination of the screening and surveillance strategies for BE. There is a need for identification of reliable clinical predictors or molecular biomarkers to risk-stratify patients who might benefit the most from screening or surveillance for BE. Finally, new therapies have emerged for the management of dysplastic BE. In this paper, we highlight the key areas of controversy and uncertainty surrounding BE. The paper discusses, in detail, the current literature about the molecular pathogenesis, biomarkers, histopathological diagnosis, and management strategies for BE.

## 1. Introduction

In the last 3 decades, the incidence of esophageal adenocarcinoma (EAC) has increased at a faster rate than any other cancer in the US and Western Europe [1–4]. Despite advances in therapies, the 5-year survival rate for EAC remains less than 15% [5]. Barrett's esophagus (BE), a condition in which the squamous epithelium of the distal esophagus is replaced by columnar epithelium with intestinal metaplasia (IM), is a well-established precursor of EAC. BE increases the risk of EAC by greater than 40-fold compared with the general population [6, 7]. Our understanding of BE has increased significantly over the past half a century. However, many aspects of the natural history and pathophysiology of BE have not been fully elucidated.

Some of the controversial areas of BE include the following:

- There is a lack of consensus regarding the definition of BE and whether IM should be a requirement for the diagnosis of BE [8–10].
- True prevalence of BE in the general population and its risk of progression to EAC remain unclear. Recent studies have suggested a lower risk of malignant transformation of BE than previously reported [11–14].
- A clear survival benefit of screening or surveillance for BE has not been demonstrated in prospective studies [8].
- There is a lack of reliable predictive biomarkers that might enable us to risk-stratify BE patients and identify those who would benefit the most from endoscopic surveillance and therapy [15].

The aim of this paper is to review the current evidence related to the definition of BE, the cancer risk of nondysplastic and dysplastic BE, screening and surveillance for BE, management of dysplasia in BE, and chemo-prevention of BE. The molecular pathogenesis, biomarkers, and histopathological diagnosis of BE will also be discussed in detail.

## 2. Historical Perspective and Definition of Barrett's Esophagus

In 1950, Norman Barrett, an Australian-born, British surgeon suggested that the finding of gastric-type mucosa in the esophagus was in most cases, due to a congenitally shortened esophagus resulting in extension of the stomach into the mediastinum. He proposed that ulcers occurring in these areas (described as *“peptic ulcers of the oesophagus”* by most pathologists of that era) were a different entity than the ulcers and strictures of the esophagus that occur as a result of acid reflux. He coined the term *“reflux oesophagitis”* for the latter disease process [17]. In 1953, Allison and Johnstone published an article titled *“The oesophagus lined with gastric mucous membrane”* to emphasize that the areas of columnar mucosa described by Barrett were actually part of the esophagus and not stomach. They proposed that ulcers in these areas be called *“Barrett's ulcers”* [18]. Seven years after his original article, Barrett agreed with Allison and Johnstone and suggested that these findings be called the *“lower oesophagus lined by columnar epithelium”* [19]. With time, the replacement of the normal squamous lining of the esophagus by columnar epithelium became known as *“Barrett's oesophagus” * [20].

In North America, BE is defined as a change in the distal esophageal epithelium of any length, recognized as columnar-type epithelium on endoscopy, and confirmed to have IM by biopsy of the tubular esophagus [8, 21]. However, differences of opinion surround this definition. There is a lack of consensus regarding the precise anatomical landmarks defining the distal limit of esophagus (gastroesophageal junction (GEJ)). According to one school of thought, the GEJ is at the proximal aspect of gastric mucosal folds. However, these landmarks can shift during phases of respiration, distension of the stomach and esophagus, and gut peristalsis [22]. Others consider GEJ as the distal end of the palisade vessels in the lamina propria of the esophagus. This landmark can be obscured by pathology in the distal esophagus and lends itself to interobserver variability [23, 24]. Three types of columnar epithelia can be seen on biopsies from Barrett's like mucosa: (a) cardia type metaplasia composed of mucin secreting glands, (b) gastric-fundus-type metaplasia comprising of parietal cells, chief cells, and mucus secreting cells, and (c) specialized IM, containing predominantly goblet cells. The first 2 types can be indistinguishable from the gastric mucosa, unless the biopsy specimen contains esophageal submucosal glands or islands of squamous epithelium [25]. If cardia- or fundic-type mucosa are obtained from the biopsies from the distal esophagus, the possibility of inadvertent sampling from the stomach should be considered. Conversely, IM can be seen in gastric biopsies due to *Helicobacter pylori* infection. To add to the complexity of this issue, studies have shown that 10–15% of normal population can have IM in the gastric cardia or GEJ [26–28]. Therefore, even the presence of IM does not completely rule out sampling error [28].

If predisposition to EAC is an essential tenet of BE, do all of the 3 types of columnar metaplasia described above increase the risk of EAC? There is robust scientific evidence that IM increases the risk of EAC [3, 29]. Some studies have suggested that cardia-type (nongoblet) metaplasia may also predispose to EAC, perhaps to a lesser extent [30, 31]. However, the true population-based risk of progression of nongoblet metaplasia to EAC is not known. There is scant scientific evidence at this time to include nongoblet metaplasia in the definition of BE.

There is also a lack of consensus among various professional organizations whether goblet metaplasia, that is, IM, should be a requirement for diagnosis of BE (also see related discussion below, in the section on histopathology). The *British Society of Gastroenterology *(*BSG*) no longer considers IM as a diagnostic criterion for BE [9]. Experts in favor of this school of thought contend that the yield of IM drops as the length of columnar lining and number of biopsies taken decrease [32, 33]. Multiple biopsies must be obtained to adequately assess for IM in patients with >1 cm of columnar lined esophagus [34]. Several arguments can be made in favor of requiring IM for a diagnosis of BE. Studies suggest that a diagnosis of BE may have a negative impact on overall quality of life of the patients. Patients with BE tend to overestimate their risk of cancer, and this leads to higher utilization of healthcare resources. A diagnosis of BE can result in higher health insurance premium and difficulty in obtaining health insurance [35–37]. The *American College of Gastroenterology *(*ACG*) and the *American Gastroenterological Association *(*AGA*) recommend documentation of IM for a diagnosis of BE [8, 21].  Table 1 summarizes definition of BE proposed by major professional organizations.

**Figure 1:**
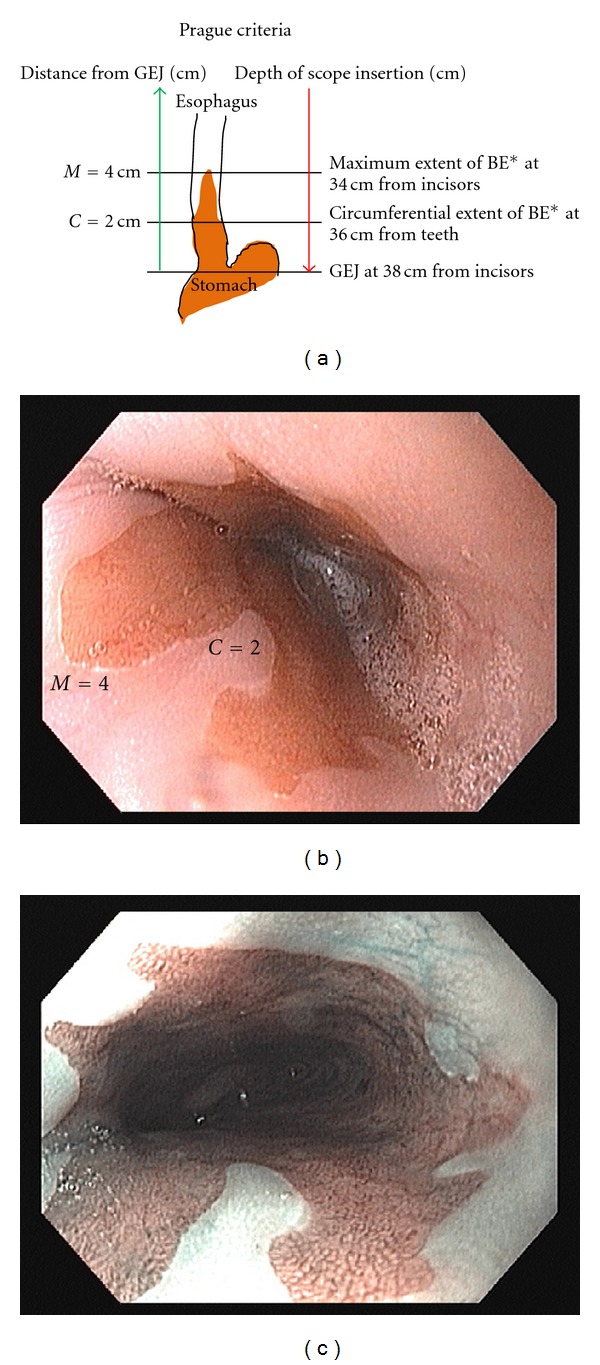
(a) Schematic representation of the Prague criteria for endoscopically suspected esophageal columnar metaplasia/Barrett's esophagus, Step 1: recognize the presence of hiatal hernia; Step 2: identify GEJ and record depth of scope insertion; Step 3: recognize suspected BE mucosa above the GEJ; Step 4: record the depth of scope insertion at the most proximal circumferential extent of BE; Step 5: record the depth of scope insertion at maximum extent of BE; Step 6: subtract the depth of insertion for circumferential and maximum extents from the depth of scope insertion at the GEJ to calculate C and M, respectively. *Endoscopically suspected columnar mucosa. Adapted from [16]. (b) endoscopic image of Barrett's esophagus for circumferential (C) and maximum (M) extent of columnar mucosa, corresponding to the schematic representation shown in Figure 1(a), (c) another endoscopic image of BE using narrow-band imaging (NBI). NBI is a high-resolution endoscopic tool that enhances mucosal surface details without the need for special dyes (electronic chromoendoscopy).

Historically, BE has been classified into short-segment (<3 cm) and long-segment BE (≥3 cm), based on the length of metaplastic epithelium seen on endoscopy [38]. A more recent classification takes into consideration the circumferential (C) and maximum (M) extent of the endoscopically visualized BE, above the GEJ. This system is called the Prague “C” and “M” criteria (Figures 1(a), 1(b) and 1(c)). The original C&M criteria were developed by an international working group using videoendoscopic recordings in 29 patients. To validate these standardized criteria, a separate panel of 29 expert endoscopists scored these recordings. The overall reliability coefficients (RCs) for the assessment of C and M extent were 0.95 and 0.94, respectively. The RCs for recognizing the location of the GEJ and the diaphragmatic hiatus were 0.88 and 0.85, respectively [16]. A recent multicenter study of gastroenterology trainees demonstrated correlation coefficients of 0.94 and 0.96, respectively, for assessment of the C and M extent of the endoscopically visualized BE. The correlation coefficients for recognition of GEJ and diaphragmatic hiatus were 0.92 and 0.90, respectively [39]. Kinjo et al. prospectively compared endoscopic BE diagnoses using the Japanese criteria (GEJ defined as the distal limit of the longitudinal or palisade vessels in the lamina propria of the esophagus) and Prague C&M criteria in 110 Japanese patients. A higher proportion of patients were diagnosed with endoscopic BE using the Japanese criteria (39%) compared with C&M criteria (26%) (*P* = 0.044). The GEJ identification rates were also higher using the Japanese criteria than with C&M criteria (95% versus 86% (*P* = 0.039)). The authors concluded that, in the Japanese population, the Japanese criteria may be more suitable for the diagnosis of endoscopic BE and for recognition of GEJ than the C&M criteria. The important limitations of this study are that only 2 endoscopists interpreted the findings, and the study was not designed to assess interobserver correlation for the 2 criteria [40]. No formal studies have evaluated the “clinical benefit” of using any standardized classification for the assessment of the endoscopic extent of BE segment. However, data suggest a direct relationship between the endoscopic extent of BE and the probability of finding IM on histology. Greater BE surface area may translate into greater cancer risk [29, 41]. Major professional societies recommend that information about the extent of metaplasia should be recorded using standardized methods [8, 21]. 

## 3. Epidemiology and Clinical Features

BE is usually detected on upper endoscopy for various indications, during the 6th decade of life or later [51, 52]. The male-to-female ratio for BE is approximately 2 : 1 [53]. White subjects have a 4–6 times higher incidence of BE compared to black subjects [2, 12, 51, 54]. Patients with chronic heart burn are 6–10 times more likely to have BE than those without heartburn [55–57]. A high body mass index and a centripetal distribution of body fat also increased the risk of BE in multiple studies [58–61]. Data suggest that *Helicobacter pylori *infection, red wine, and diet rich in fruits and vegetables may have protective effect against BE [62–67].

The reported prevalence of BE in different studies has varied widely, depending on the population studied and the definition used. A study of 733 unselected autopsies from Olmsted county, MN, USA, estimated the prevalence of BE to be 376 per 100,000 population [68]. A retrospective analysis of a US rural white population in the Marshfield Epidemiologic Study Area (MESA), estimated the prevalence of endoscopically and histologically confirmed BE to be 261.8 per 100,000 persons [69]. BE was diagnosed in 6.8% patients in a study of 961 US patients who were scheduled for a colonoscopy and had no prior history of an upper endoscopy. However, the study population comprised of predominantly white males [12]. There is a lack of large population-based studies on the prevalence of BE in the United States. A Swedish study reported the prevalence of BE to be 1.6%, when both endoscopic and histological criteria were used to define BE [70].

The presence of columnar mucosa in the distal esophagus does not cause symptoms per se. The symptoms in these patients are mostly related to sequelae of long-standing gastroesophageal reflux disease (GERD) (e.g., esophagitis, peptic stricture, etc.). Absence of chronic reflux symptoms does not exclude the possibility of BE. Indeed, in the Swedish study, about 44% of the patients found to have BE did not report significant symptoms of heart burn and dyspepsia [70]. BE is seen in about 10–15% of the patients undergoing upper endoscopy for chronic GERD, and in 5.6% of those without chronic reflux symptoms [71]. The frequency of long-segment BE is 3–5% and short-segment BE is 10–15% among patients with chronic GERD [72].

## 4. The Pathogenesis of Barrett's Metaplasia 

### 4.1. Cell of Origin

A very important step in understanding the development of BE and its progression to EAC is identifying the cell of its origin. Unfortunately, the cell of origin in BE is not known. There exist currently 2 conflicting hypotheses. The first possibility is that BE arises from a stem cell. To complicate this question further, the origin of this putative stem cell is also up for debate. It is possible that the BE stem cell originates from the stratified squamous epithelium compartment, the submucosal glands of the distal esophagus, bone-marrow-derived stem cells (BMDCs), or stem cells located in the proximal stomach [73–77]. Search for a putative BE stem cell is hindered by the lack of knowledge regarding stem cells of the esophagus. To date, only one group has identified a population of squamous epithelial-derived cells enriched for stem cell properties which can be isolated by flow cytometry sorting for CD34, a marker for stem cells in other tissues [78].

A second hypothesis for the cell of origin of BE is that of a differentiated esophageal keratinocyte. One presumption for this hypothesis would be that the squamous epithelium would undergo at least a partial dedifferentiation. Recent work to generate pluripotent-induced progenitor stem cells (iPS) by introduction of c-myc, KLF4, Sox2, and Oct3/4 into adult fibroblasts has lent credence to this possibility [79]. In fact, several of these genes have been demonstrated to play a role in esophageal and BE biology. Sox2 has been determined to be a lineage-specific transcription factor in the esophagus specifying squamous cell differentiation [80]. KLF4 and c-myc expressions are upregulated in Barrett's esophagus and may contribute to a transdifferentiation process [81, 82]. Further work on stem cells and the molecular pathways involved in the transdifferentiation process of BE will be necessary to address the cell of origin question.

### 4.2. Reflux Exposure and Inflammation

One of the most strongly associated clinical symptoms for the development of BE is the recurrent reflux of acid and bile salts. Epidemiologic data suggests that GERD is important for the development of BE [83]. Approximately 13% of patients that have been diagnosed with GERD develop BE [84]. These data suggest a role for GERD in the development of BE. In fact, surgical anastomosis of the esophagus to the small intestine by either joining to the duodenum (EDA or EGDA models) or jejunum (EJA) in rats has verified this hypothesis [85–87].

The chronic reflux associated with GERD induces a significant amount of epithelial damage and a commensurate inflammatory response. Research over the last several decades has begun to illustrate the role inflammation plays in many disease processes, and this is likely true with the development of BE and esophageal adenocarcinoma. Work has focused on cytokines secretion in BE, signaling events induced in the esophageal epithelium in response to GERD, and the accompanying inflammation that may be responsible for the metaplastic process in BE cells. Acid and bile salt exposure may impair the barrier function as well [88]. This loss of barrier function and damage to the epithelium may be the precipitating events for the initial inflammatory response. This induces a Th2 inflammatory response replete with the expression of IL1*β*, IL4, IL6, IL8, TNF*α*, and IFN*γ* [89]. This type of a response has been associated with tumor formation and progression by recruiting immature myeloid cells, tumor-associated macrophages, and neutrophils [90–94]. This type of immune response may have a similar role in triggering signaling events that result in metaplasia of the epithelium.

Several signaling pathways associated with inflammation have been implicated in BE. Cyclooxygenase (Cox) gene expression appears to be induced by acid reflux. In fact, its expression level is positively correlated with the degree of acid reflux which occurs in a given patient. Interestingly, Cox2 expression increases as BE progresses to EAC, suggesting that Cox2 may functionally contribute to the pathogenesis of EAC [95]. Indeed, epidemiologic data demonstrate that anti-inflammatory treatment decreases cancer risk due to Cox2 inhibition [96, 97].

In addition to Cox genes expression, NF*κ*B expression has also been implicated in the initial stages of the development of BE. Induction of NF*κ*B in esophageal cells subsequent to inflammation-inducing reflux many have effects within the epithelium including the induction of cell survival pathways as well as the transcription of target genes involved in the development and progression of BE. Indeed, NF*κ*B has been demonstrated to be a direct transcriptional regulator of the caudal, homeodomain-containing transcription factors, Cdx1 and Cdx2 [98, 99]. Cdx genes have been implicated in the earliest stages in the transdifferentiation process of BE [81].

## 5. Biomarkers of Barrett's Esophagus

Genome-wide examination of BE lesions had been performed in order to characterize BE for gene expression changes when compared to normal esophagi, EAC, gastric epithelia, gastric cancers, small intestinal epithelia, and colonic epithelia. The use of microarray expression analyses has generated a large amount of data characterizing the differences between these tissues. Many of the arrays have identified genes that function in cell growth and proliferation, apoptosis, cell metabolism, lipid metabolism, catabolism, cell adhesion, signal transduction, migration, stress response, cell adhesion, signal transduction, and transcription factors [100–106]. While these lists cover a wide array of cellular functions, many of these categorizations seem logical based on what is currently known about BE.

One interesting publication describes the identification of two distinct subcategories of BE dubbed as BE1 and BE2 [102]. BE1 identifies a group similar to that of normal stomach mucosal samples and includes several tumor suppressor genes. BE2 identifies a group that may lie closer to EA than does BE1. BE2 gene expression contains genes that have been either overexpressed in cancers such as carboxylesterase 2, galectin-4, glycoprotein A33, and LI-cadherin. These classifications of different BE samples are intriguing and may be reflected in two other microarray studies. One study from Barrett et al. subclassified BE samples into several categories including their own BE1 group which had a higher correlation coefficient with squamous samples while sample their BE4 group had a higher correlation with their gastric samples [100]. A third study from the University of Pennsylvania suggested that two populations of BE may exist [81]. Principle component analysis placed BE samples into 2 groups spatially that were distinct from each other yet closely related based on global gene analysis. A meta-analysis of all BE samples to determine if classifications extend beyond these data sets would be very informative. Differences between BE groups may identify biomarkers that would predict progression. Several microarray expression analyses have begun to identify genes that may predict the progression of BE to EAC [103, 104, 106]. These genes include MMP7, CXCL3, GATA6, HoxB7, and SPRR3. Pepe et al. have defined five phases of biomarker development to establish a gene as a *bone fide* biomarker of disease [107]. Few biomarkers have been evaluated for risk stratification using this systematic approach in BE. Unfortunately, most of the microarray data published to date have lacked followup work to identify those genes contained within the data sets that contribute to the cause of BE or its progression to EAC or in the alternative to serve as true biomarkers for disease progression.

## 6. Signaling Pathways

As had been previously discussed, many factors such as acid, bile salts, and inflammation are likely to contribute to the pathogenesis of BE. What remains less well defined are the intracellular signaling events responsible for the transition to BE which is likely due to several signaling pathways altering the differentiation of the epithelium of the esophagus. As a result, an approach whereby several signaling pathways are manipulated at one time is needed. As previously mentioned, Cdx gene expression has been detected in BE and is likely a result of NF*κ*B expression subsequent to the inflammatory response elicited by acid reflux. Indeed, Cdx gene expression is one of the most likely candidates for transcription factors that induce intestinal metaplasia due to its well-established role in embryonic intestine development. Additional signaling events that may induce Cdx expression are retinoic acid (RA) and hedgehog signaling [108–112].

Another transcription factor, c-myc, is important for the development of BE [81, 113]. Interestingly, c-myc expression increases in dysplastic lesions and reaches its highest expression in EAC. Also, c-myc activation has been implicated in BE by microarray analysis [81]. To test whether c-myc was involved in BE, c-myc was coexpressed with Cdx1 in squamous esophageal cell lines and induced several genes expressed in BE, suggesting a partial shift in differentiation.

## 7. Histopathology Diagnosis and Dysplasia Grading

### 7.1. Definition: Intestinal Metaplasia or No Intestinal Metaplasia

As discussed above, the definition of BE varies worldwide [114]. Countries such as USA require the presence of goblet cells (IM), whereas England and Japan accept any endoscopically visible columnar metaplasia as sufficient to define BE. The requirement of IM has been supported by studies that have claimed IM to be a prerequisite for the development of adenocarcinoma [115, 116]. More recently, there has been a suggestion to move away from this view [10]. Gatenby et al. evaluated clinical followup for 934 patients, including 322 with intestinal metaplasia and 612 with nongoblet columnar cell metaplasia of the distal esophagus, and found no difference with respect to dysplasia and/or cancer incidence between the two groups (19.8% versus 15.2%, resp.), suggesting that nongoblet glandular epithelium in the distal esophagus may also be at risk for neoplastic transformation [117]. As suggested by DeMeester, a practical approach may be to consider that patients with ≥3 cm of columnar-lined esophagus nearly always either have or will develop IM, and these patients should be considered to have BE along with those with shorter lengths of columnar mucosa who show IM on biopsy [118].

Goblet cells may be identified on routine histological stains (hematoxylin and eosin, H&E), although many institutions routinely employ PAS-Alcian blue stains to highlight acidic mucin in goblet cells (blue by Alcian blue at pH 2.5) against a backdrop of gastric neutral mucin (magenta caps by PAS). This stain is helpful in differentiating true IM from so-called columnar blue cells or pseudogoblet cells, which are gastric foveolar cells that have been “injured” by acid reflux disease. The Alcian blue stain also helps in easy identification of IM and saves time in evaluating a biopsy. Another clue to the presence of IM is the presence of so-called “multilayered epithelium” which is characterized by the presence of an overlay of columnar cells over basal layers of squamous cells. In their study of 17 cases, Glickman et al. found that glandular cells of multilayered epithelium express different mucin types with similar frequencies to IM and also frequently express CDX2 and MUC2, similar to goblet cells that occur in the esophagus. They proposed that multilayered epithelium is a precursor to intestinal metaplasia; presence of multilayered epithelium should prompt a more detailed search for goblet cells [119].

The IM of gastric cardia mucosa does not equate to BE, since BE has to involve the tubular esophagus. Also, IM of gastric cardia may be less likely to progress to dysplasia than true BE [120]. Thus a diagnosis of BE cannot be made histologically when the exact site of biopsy of the metaplastic fragment is not known. BE is a clinicopathologic diagnosis and the biopsy must be from an endoscopically visualized abnormality in the tubular esophagus. IM may be either “complete” (when goblet cells are accompanied by absorptive and/or Paneth cells) or “incomplete” (absence of absorptive and/or Paneth cells). Conceptually speaking, incomplete IM is less differentiated and therefore more likely to be a dysplasia precursor. In practice, incomplete and complete IM may exist adjacent to each other, and their identification may purely be a result of sampling. Hence, subtypes of IM are not generally mentioned in pathology reports.

### 7.2. Dysplasia: Negative and Indefinite

**Figure 2:**
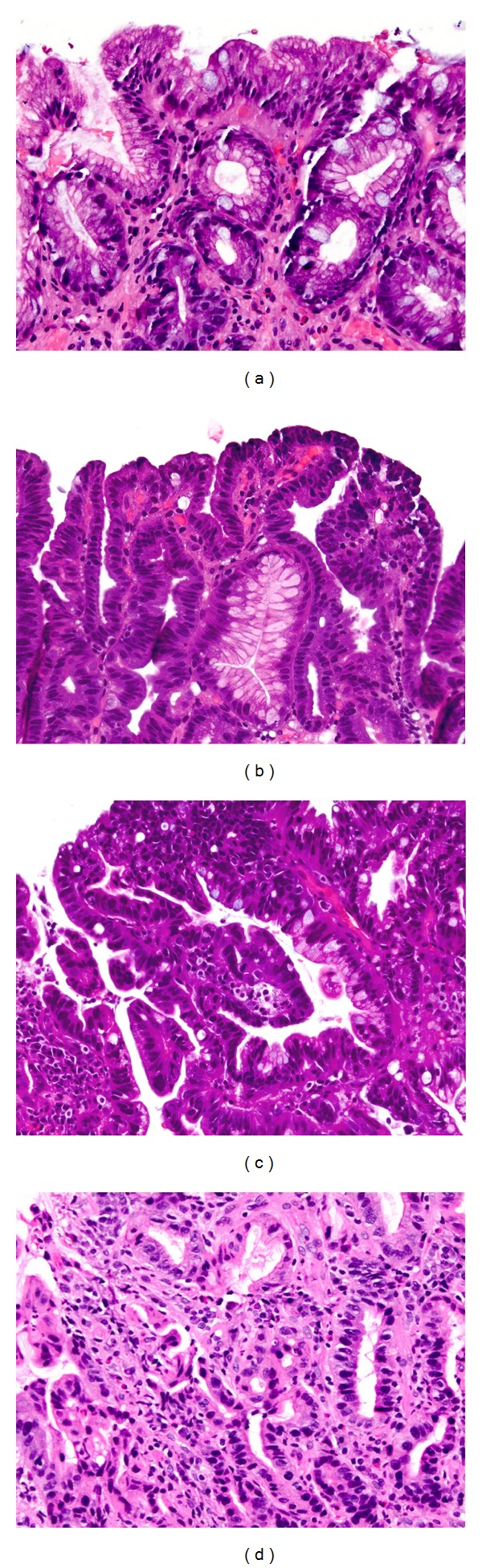
(a) Intestinal metaplasia is defined by the presence of goblet cells distended with mucin. In this photomicrograph there is no dysplasia, as evidenced by presence of surface maturation. Surface epithelial cells show uniform mucin caps and well-polarized nuclei. (b) Low-grade dysplasia of the intestinal type is characterized by hyperchromatic elongate nuclei that are seen in both the crypts and the surface epithelium (i.e., loss of surface maturation). (c) Presence of glandular crowding, nuclear stratification, and loss of nuclear polarity signifies high-grade dysplasia. (d) Glandular complexity, budding, and presence of incomplete glandular profiles are evidence of lamina propria invasion (intramucosal carcinoma).

All biopsies for BE diagnosis and surveillance should include a qualifier regarding presence or absence of dysplasia. Dysplasia is assessed in columnar mucosa, preferably away from squamous mucosa, and where the surface lining is intact. Biopsies are categorized as being “negative for dysplasia” if the cells show maturation towards the surface in the form of decreasing nuclear size, decreasing nuclear hyperchromasia, increasing cytoplasmic volume and a mucus cap on surface cells (Figure 2(a)). In some cases, there may be some changes that are deemed insufficient to characterize as dysplasia, and these are categorized as “indefinite for dysplasia”. Cases that are classified as being indefinite for dysplasia are either those with minimal to mild cytologic atypia or those that have more than mild cytologic atypia but are accompanied by significant inflammation, raising a possibility of atypia reactive to the inflammatory response. These cases need rebiopsy after control of inflammation. Reactive changes tend to be more diffuse rather than abrupt, the latter being a feature of dysplasia. The category of “indefinite” should not be used to downgrade dysplasia, but rather to identify cases that may need followup biopsies.

### 7.3. Dysplasia: Intestinal Type

Most dysplasias occurring in a setting of IM are of the “intestinal type.” Criteria used to classify dysplasia in this setting are similar to what has been used in colonic adenomas. This pattern of dysplasia is therefore morphologically recognized by the presence of hyperchromatic elongate nuclei, with lack of surface maturation and presence of nuclear crowding, such that the surface epithelium appears generally similar to the glands at the depth. In low-grade dysplasia (LGD), architecture is by and large preserved, true nuclear stratification is absent, and, as a rule, nuclear polarity is maintained (Figure 2(b)). Changes are usually patchy or focal with relatively abrupt transformation between dysplastic and nondysplastic foci, although this transition may not always be evident.

High-grade dysplasia (HGD) is usually identifiable at low-power examination by architectural abnormalities such as glandular crowding, branching, and complexity. Nuclear atypia is also more pronounced, with increasing nuclear stratification and nuclei reaching the surface of the lining cells, without a clear cytoplasmic zone at the apex (Figure 2(c)). There is chromatin smudging, increasing nuclear irregularity, and presence of surface mitoses. Loss of nuclear polarity is the single most helpful feature to identify HGD at the cytologic level. In a setting of HGD, presence of cribriform (“back to back”) glands, luminal necrosis, glandular budding, and incomplete glands may indicate intramucosal carcinoma (Figure 2(d)) [121].

### 7.4. Dysplasia: Basal Crypt Dysplasia and Foveolar Dysplasia

Basal crypt dysplasia was recently described as another variant of dysplasia. This entity is recognized by the presence of dysplasia-like atypia affecting the deep/basal crypts with the presence of surface maturation. Lomo et al. consider this to be true dysplasia in view of the presence of molecular abnormalities (significantly increased rate of 17p (TP53) LOH and aneuploidy), and a high association with conventional dysplasia and/or adenocarcinoma, despite the morphologic appearance of surface maturation. The clinical, pathologic, immunohistochemical, and molecular abnormalities were similar in cases showing low-grade and high-grade histological changes in the basal crypts, except that high-grade cases tended to occur in older patients [122]. Presently, there are no clear guidelines on followup of patients with basal crypt dysplasia. Many pathologists tend to diagnose these cases as indefinite for dysplasia with a comment that dysplasia is restricted to the basal crypts.

Gastric foveolar-type dysplasia is another distinct variant of dysplasia in BE. This dysplasia is similar to the so-called type II dysplasia that is known to occur in the stomach [123, 124]. The diagnosis of gastric foveolar-type dysplasia in BE can be challenging, since there are no standardized diagnostic or grading criteria. Mahajan et al. evaluated the prevalence, morphology, and natural history of gastric foveolar-type dysplasia in a cohort of 200 BE patients. The prevalence of gastric foveolar-type dysplasia was 15% at the patient level and 20% at the biopsy level. Unlike intestinal-type dysplasia, gastric foveolar-type dysplasia uniformly showed nonstratified, basally oriented nuclei, explaining the difficulty in its recognition. This uniformity therefore precludes the use of loss of nuclear polarity in identifying HGD within this group. Rather, the most important feature permitting a diagnosis of high-grade gastric foveolar-type dysplasia was significantly increased nuclear size (to approximately 3-4 times the size of a mature lymphocyte). Other features in high-grade lesions included villiform architecture, crowded, irregular glandular architecture, eosinophilic and oncocytic cytoplasm, prominent nucleoli, and mild nuclear pleomorphism. During followup, 64% (7 of 11) of patients with pure gastric and 26% (5 of 19) with mixed gastric and intestinal dysplasia underwent neoplastic progression [125].

### 7.5. Interobserver Variation in Dysplasia Grading

Given the subjectivity in identifying and grading dysplasia, it is not surprising that there is a fair amount of interobserver variability in the pathologic evaluation of BE biopsies. In a study by Kerkhof et al., general pathologists were found to overdiagnose HGD [126]. Nearly 40% of patients who were initially diagnosed with HGD by a general pathologist were downgraded (11% no dysplasia, 12% indefinite for dysplasia, 16% LGD) when the samples were reviewed by three experienced gastrointestinal (GI) pathologists. Downs-Kelly et al., however, reported overall poor interobserver reproducibility even among GI pathologists who see a high volume of Barrett's cases, calling into question treatment regimens based on the assumption that HGD, intramucosal adenocarcinoma, and submucosal adenocarcinoma can reliably be distinguished in biopsy specimens [127]. Montgomery et al. evaluated reproducibility of diagnoses among expert GI pathologists and found that reproducibility improved only after a consensus meeting to decide on criteria to be used. Interobserver variation was better for clinically relevant separation into two groups (BE indefinite and LGD; versus HGD and carcinoma), than into four groups (BE; indefinite and LGD; HGD; carcinoma). Agreement was least for distinction between indefinite and low-grade categories [128]. Followup data collected on 138 cases from the above study were separately analyzed. Dysplasia grade on initial biopsy correlated significantly with progression to invasive carcinoma. Using the initial submitting diagnoses, carcinomas were detected on followup in 18% of indefinite, 15% of LGD, and 61% of HGD cases. When majority diagnoses (among 12 GI pathologists) were used for analysis, carcinomas were detected in 14% indefinite, 20% of LGD, and 60% of HGD cases. There were 39 cases without a majority diagnosis, among which carcinomas were detected in 43% cases with an average score between indefinite and LGD and 70% cases with an average score between LGD and HGD [129]. Interestingly, interobserver reproducibility for basal crypt dysplasia has been reported to be better than conventional LGD but poorer than that for HGD and BE without dysplasia [130]. Notwithstanding the relative lack of reproducibility, it is opined that, in general, pathologists perform extremely well when diagnosing lesions of the highest and lowest risk [131].

### 7.6. Dysplasia: Role of Immunohistochemistry

Studies have also addressed the utility of immunohistochemical markers in identifying and grading dysplasia. Immunohistochemistry for p53 has been reported to assist in diagnosis in difficult cases [132]. Immunohistochemistry for p53 and Ki67 has also been reported to correlate with the severity of dysplasia in assessing Barrett's biopsies [133, 134]. In another study of 86 biopsies, protein overexpression of *β*-catenin helped diagnose LGD, whereas overexpression of cyclin D1 and p53 discriminated HGD from LGD [135]. However, most pathologists rely on routine stains and morphology to diagnose and grade dysplasia and immunohistochemistry is rarely, if ever, used in routine day-to-day practice in most centers.

## 8. Risk of Progression of Nondysplastic Barrett's to Esophageal Adenocarcinoma

Initial studies observed that the risk of progression of BE to EAC ranged between 2 and 4% per year [136–138]. Shaheen et al. noted that these studies might have overestimated the risk due to publication bias in favor of smaller studies reporting relatively higher incidence [139]. Data from subsequent studies showed a lower risk. A meta-analysis of 41 studies with a total followup of 36,635 person years showed 0.7% annual risk of progression from BE to EAC. There was no evidence of significant geographic variation between US, UK, and Europe. There was a trend towards decreased risk of EAC in short-segment BE [29]. Another exhaustive review of forty-seven studies reported a pooled annual estimate of 0.61% for cancer incidence in patients with BE. When early-incident cancers were excluded, the risk decreased to 0.53% per year. When both early incident cancers and HGD at baseline were excluded, the risk was noted to be 0.41% per year [3]. 

More recent studies have suggested a lower risk of progression of BE to EAC. Bhat et al. analyzed data on 8522 patients with BE from the Northern Ireland Barrett's esophagus register (NIBR). The mean followup was 7 years. Incidence of EAC, carcinoma of the gastric cardia, and HGD was reported to be 0.22% per year in the entire study cohort. Of note, this study used the British definition of BE whereby IM was not required for diagnosis of BE. The incidence of EAC, carcinoma of the gastric cardia, and HGD in patients with IM on index biopsies (criteria used in the US) was 0.38% per year versus 0.07% in those without IM (HR = 3.54, 95% CI = 2.09 to 6.00, *P* < 0.001). The risk was significantly higher in males versus females (HR = 2.11) [7]. A recent study from Denmark reported the incidence of EAC to be 0.29% per year when 11,028 patients with BE were followed for 67,105 person-years. When the EAC cases diagnosed within first year of followup were excluded, the overall annual incidence decreased to 0.12%. The incidence of EAC was 0.1% per year among patients with NDBE on index endoscopies [14].

## 9. Risk of Progression of Nondysplastic Barrett's to Adenocarcinoma of the Gastric Cardia 

Data suggest that chronic reflux and BE increase not only the risk of EAC but also that of adenocarcinoma of the gastric cardia. Ruol et al. compared the epidemiological, clinical, and pathological characteristics of 26 patients with EAC in BE and 16 patients with adenocarcinoma of the gastric cardia. They observed that IM was present in the mucosa adjacent to 25/26 (96%) patients with EAC in BE, and 11/16 (69%) patients with adenocarcinoma of the gastric cardia. Authors concluded that IM may be a common precursor of adenocarcinoma in BE and adenocarcinoma of the gastric cardia [116]. In a retrospective study of the pathology specimen of 100 patients who underwent esophagectomy for adenocarcinoma of the esophagus, cardia, or proximal stomach, specialized IM was identified in the resected specimen in 13/31 (42%) of patients with adenocarcinoma of the cardia as opposed to 1/21 (5%) in those with adenocarcinoma of the proximal stomach [140]. In the Northern Ireland Barrett's esophagus registry, 16 incident cases of adenocarcinoma of the gastric cardia were reported during a mean followup of 7 years in 8522 patients [7].

## 10. Risk of Progression of Dysplastic Barrett's to Esophageal Adenocarcinoma

### 10.1. Risk of Progression of Low-Grade Dysplasia to Esophageal Adenocarcinoma

Reported incidence of EAC in patients with LGD has ranged between 0.6% and 13.4% per year, with most studies suggesting the risk to be less than 1% per year.  Table 2 summarizes the key findings of the studies evaluating the risk of progression of LDG to EAC [7, 14, 43–46]. 

### 10.2. Risk of Progression of High-Grade Dysplasia to Esophageal Adenocarcinoma

Patients with HGD are at high risk of progression to EAC [141].  Table 3 summarizes the key findings of the studies that have evaluated the risk of progression of HGD to EAC [47–49]. Based on the current literature, the risk of EAC in HGD may be more than 10% per patient-year [49, 142, 143]. The extent of HGD may also be an important determinant of malignant potential. Diffuse HGD was noted to have a 3.7-fold higher risk of progressing to EAC compared with focal HGD in a retrospective cohort study [144].

## 11. Screening for Barrett's Esophagus

Approximately 40% of adults in the US experience symptoms of heart burn at least once a month and about 20% report these symptoms once a week [145]. Considering, chronic GERDs is the strongest risk factor for BE, theoretically a large proportion of adult US population would be eligible for screening for BE based on this screening criteria. However, about 40% of the patients diagnosed with EAC do not report chronic GERD symptoms [6]. Thus, if chronic GERD were to be used as a screening criterion, close to half of the targeted population will be missed. Despite a rapid increase in the incidence of EAC in the past 3 decades, the absolute number of EAC cases diagnosed annually in the United States is approximately 10,000. This is a small percentage of patients with chronic GERD. Even when diagnosed with BE, a vast majority of these patients will not develop EAC during their lifetime [47]. Multiple studies have shown that a diagnosis of BE does not necessarily translate into an increased all-cause mortality compared with age- and sex-matched controls. A vast majority of patients with BE die due to causes other than esophageal cancer (e.g., cardiovascular disease) [45, 146, 147]. There is a lack of data showing a clear benefit of screening for BE. The current position of the *AGA* is that *“inadequate evidence exists to endorse endoscopic screening for BE based solely on the presence of GERD symptoms.”* The decision regarding screening should be individualized after discussion about the benefits and limitations of screening with the patient [8]. The *ACG* guidelines state that *“screening for BE in general population cannot be recommended at this time.”* The use of screening in high-risk population remains to be established and should therefore be individualized [21]. The *American Society of Gastrointestinal Endoscopy* (ASGE) guidelines consider an initial screening endoscopy to be appropriate in select patients with frequent (several times per week), chronic, long-standing GERD (>5 years), who are white, males, aged >50 years, and those with nocturnal heart burn. No further screening is needed if the initial esophagogastroduodenoscopy (EGD) is negative for BE [50]. The *BSG *and the *French Society of Digestive Endoscopy *(*FSDE*) do not recommend routine screening for BE [9, 42, 71].  Table 4 summarizes guidelines for screening for BE by major professional organizations.

## 12. Surveillance of Patients with Barrett's Esophagus

Multiple studies have demonstrated that cases of EAC diagnosed as a result of endoscopic surveillance are associated with an earlier stage of disease and improved survival compared with clinically diagnosed cases [148–152]. These data present a rationale for surveillance of patients with BE. However, several arguments can be made to the contrary. There are no prospective data showing survival advantage with surveillance. The observational studies demonstrating benefit of surveillance are limited by design flaws and lead time bias. A vast majority of patients diagnosed with EAC do not carry a prior diagnosis of BE [147, 151, 153, 154]. Current surveillance methods have limitations. Our ability to detect dysplasia is highly dependent on adherence to rigorous biopsy protocols. One such protocol, called the Seattle Protocol, recommends 4 quadrant biopsies every 1-2 cm throughout the length of columnar-lined esophagus and separate biopsies from other areas of mucosal abnormalities such as ulcers or nodules [155, 156]. Studies have shown that overall adherence to validated biopsy protocols among gastroenterologists is suboptimal and decreases even further as the length of columnar lined mucosa increases [156, 157]. Interobserver variability among expert GI pathologists in diagnosing the presence and degree of dysplasia has been demonstrated in multiple studies [128]. As noted above, the overall risk of EAC in patients with BE is low, with recent studies showing annual incidence much lower than 0.5% [14]. Despite limitations of the scientific evidence, most professional societies recommend endoscopic surveillance for patients with BE. As discussed in previous sections, the risk of EAC increases as NDBE progresses in a sequential manner to LGD and HGD. The frequency of surveillance is therefore based on the grade of dysplasia.

The *ACG* recommends that all patients with documented BE should be assessed for surveillance. The decision should take into consideration patient's age, likelihood of survival over the next 5 years, and willingness to adhere to the surveillance program after understanding the risks, benefits, and limitations of surveillance. In order to avoid interference due to inflammation from reflux esophagitis, patients' symptoms of GERD should be controlled with proton pump inhibitors (PPIs). Surveillance EGD should include 4 quadrant biopsies from every 2 cm of Barrett's mucosa. Separate biopsies must be taken from other areas of mucosal abnormalities such as ulcers or nodules. Patients with BE with no evidence of dysplasia on index endoscopy should undergo a repeat endoscopy with protocol biopsies within 1 year. If no dysplasia is found, surveillance should be done every 3 years. Finding of LGD should be confirmed by an expert GI pathologist. If LGD is confirmed, an upper endoscopy should be repeated 6 months later to rule out a higher grade of dysplasia. If this is not found to be the case, endoscopy should be done annually until no dysplasia is seen on 2 consecutive endoscopies. Mucosal abnormalities (e.g., nodules) in a background of HGD should undergo endoscopic mucosal resection (EMR) [21]. Finding of HGD should also be confirmed by an expert GI pathologist, and an upper endoscopy with biopsies should be repeated within 3 months. Most experts agree that finding of HGD should trigger intensive surveillance endoscopies every 3 months and a detailed discussion with the patient about endoscopic therapeutic options (see below). Mucosal abnormalities (e.g., nodules) in a background of HGD should undergo endoscopic mucosal resection (EMR) [21].

Other major gastroenterology organizations in the US endorse most components of ACG guidelines on surveillance for BE.  Table 5 summarizes the guidelines for surveillance of BE by major professional organizations in the United States. 

## 13. Management of Barrett's Esophagus 

### 13.1. Management of Nondysplastic Barrett's Esophagus

All patients with BE should be on acid-suppressive therapy to control symptoms related to GERD. However, there is no convincing evidence that acid-suppressive therapy or antireflux surgery reverses the IM or dysplasia [8, 158]. Although endoscopic eradiation therapies (EET) have been tried successfully in NDBE, at this time most professional organizations do not recommend EET for NDBE [8, 21, 159].

### 13.2. Management of Barrett's with Low-Grade Dysplasia

The conventional approach for management of LGD has been that of careful endoscopic surveillance biopsies and EMR for all mucosal nodules or irregularities [21]. However, data from some studies showing relatively high malignant risk of LGD and growing literature on safety and efficacy of endoscopic ablation therapies has sparked a debate about the appropriate management of LGD. Those in favor of watchful waiting view the data on high malignant risk of LGD with skepticism. These studies are small in size, and most do not distinguish between prevalent LGD and incident LGD. The latter issue is important to recognize because malignant risk may be different between prevalent and incident LGD. Some of the studies have lumped together HGD and EAC as their final outcome of interest which can skew the data towards a higher risk. The important issue of interobserver variability (see above) in LGD has not been addressed in most of these studies [160]. One also needs to be cognizant of the phenomenon of regression reported in LGD [43]. It is not clear whether this occurs due to initial overdiagnosis, sampling errors, or true regression. Finally, the cost and risks of endoscopic therapies need to be weighed against the benefit of treating LGD, a condition whose malignant risk has not been clearly defined at this time [161, 162].

Endoscopic therapies for LGD have been used successfully. Those in favor of endoscopic ablation for LGD view this therapy as safe, effective, and durable. In a trial of RFA in dysplastic BE, among the patients with LGD, complete eradication of dysplasia was seen in 90.5% patients who received RFA compared with 22.7% in controls (sham procedure) (*P* < 0.001) [163]. Complete ablation of LGD and NDBE may also give the patients peace of mind, although data backing this assumption are lacking. However, radiofrequency ablation (RFA) can result in upper GI bleeding and about 6% rate of esophageal strictures [163]. Esophageal strictures and photosensitivity reaction to chemosensitizing agent were reported in 36% and 69% patients, respectively, who underwent photodynamic therapy (PDT) [142]. Some experts recommend an individualized approach based on risk startification and patient preference. Based on the available data, the ACG, AGA, and ASGE guidelines do not recommend routine endoscopic ablation for LGD at this time [8, 21, 50].

### 13.3. Management of Barrett's with High-Grade Dysplasia

Before the advent of endoscopic therapies, esophagectomy was the primary treatment option for patients with HGD. Data from older series showed up to 40% coexisting cancer in patients who underwent esophagectomy for HGD [164]. However, recent studies suggest that 12.7% prevalence of EAC in these cases may be a more accurate estimate. If patients without any endoscopically visible lesions are analyzed, the rate drops to about 3% [165]. Furthermore, even in expert hands, esophagectomy carries a mortality risk of 1–5% and morbidity risk of 30–50% [164, 166]. There are no prospective, randomized trials comparing EET directly with esophagectomy for management of HGD or intramucosal adenocarcinoma of the esophagus. However, this question has been evaluated in retrospective studies. Prasad et al. retrospectively compared data on 129 patients who received endoscopic therapy (PDT ± adjunctive EMR) and 70 patients who underwent esophagectomy for HGD at their institution. The patients in the PDT group were followed for a mean of  59 ± 2.7  months and those in the esophagectomy group for a mean of  61 ± 5.8  months. The overall mortality was similar in the 2 groups at the end of followup period (9% in the PDT group versus 8.5% in the esophagectomy group, *P* = 0.76) [167]. Another study identified 742 patients with early esophageal cancer, in a review of surveillance epidemiology and end results (SEER) database. About 13% of these patients were treated with endoscopic therapy and rest with surgical resection. The risk of esophageal cancer-related mortality was similar between the endoscopic and surgical groups (HR = 0.89; CI = 0.51 − 1.56; *P* = 0.68) [168].

EMR is an important tool in accurate staging of the disease that can in turn guide appropriate therapy. The specimens obtained from EMR are larger than the mucosal biopsies and provide a better assessment of the depth of tumor invasion into mucosa and submucosa. Larghi and colleagues performed EUS to stage the disease of 48 BE patients with biopsy-proven HGD/intramucosal carcinoma. EUS showed mucosal disease in 40 patients (25 HGD and 15 intramucosal carcinomas). Eight patients found to have submucosal invasion on EUS were excluded. EMR was done in the 40 patients with disease confined to the mucosa. Compared with EUS, the EMR upstaged the disease from HGD to intramucosal carcinoma in 6/25 (24%) patients and from intramucosal carcinoma to submucosal invasion in 6/15 (40%) patients [169]. A systematic review showed that EMR resulted in change of diagnosis in about 25% of cases when compared with mucosal biopsies (upstaging or downstaging) [170]. Role of circumferential EMR in eradication of BE with HGD has been evaluated. In one study, 12 patients with BE and HGD or intramucosal cancer underwent circumferential EMR (median of 2.5 sessions, median of 5 snare resections per session). No recurrence of BE or cancer occurred during a median followup of 9 months. Complications included esophageal strictures in 2 patients treated successfully by bougienage, and minor bleeding during 4/31 EMR sessions [171]. Other studies on circumferential EMR have reported 86–100% complete eradication rate, and recurrence of malignancy in up to 11% cases. A high stricture rate is a limitation of this technique [172, 173].

Although a variety of endoscopic ablation therapies have been studied for eradication of HGD, the best evidence is available for RFA and PDT. In a multicenter, sham-controlled trial, 127 patients with dysplastic BE were randomized to RFA (*N* = 84) or sham procedure (*N* = 43). Among patients with HGD, complete eradication of dysplasia was achieved in 81% in the ablation group compared with 19% in the control group (*P* < 0.001). One patient developed upper GI hemorrhage and 5 (6%) developed esophageal stricture in the ablation group. All complications were successfully managed endoscopically [163]. As a separate study, in the same population, subjects in the sham group were offered crossover to the RFA treatment after the completion of first 12 months. All subjects were followed for 2 years from initial enrollment. Those who had achieved complete eradication of IM at 2 years were followed for 3 additional years with annual surveillance EGD and biopsies. At the end of study period, dysplasia remained eradicated in >85% patients and intestinal metaplasia in >75% without maintenance RFA. The authors concluded that RFA offers safe, effective, and durable therapy for dysplastic BE [174].

Overholt et al. performed porfimer-PDT in 103 patients with BE and used supplemental Nd:YAG laser to ablate small areas of residual Barrett's mucosa. Mean followup for the 82 patients who completed the study was 58.5 months. About 94% (60/65) patients with HGD had eradication of dysplasia. Three (4.6%) patients developed subsquamous adenocarcinoma. Strictures occurred in 30% patients [175]. In a multicenter, randomized trial, 208 patients with HGD were randomized to receive PDT plus omeprazole (PORPDT group, *N* = 138) or omeprazole only (OM group, *N* = 70). Complete ablation of HGD was seen in 106/138 (77%) patients in the PORPDT group versus 27/70 (39%) patients in the OM group. PORPDT group experienced photosensitivity reactions in 69% patients. About 36% patients in this group developed esophageal strictures that were managed successfully with endoscopic dilatation [142].

The current *ACG* guidelines state that treatment for HGD should be individualized. Patient should be presented the options of EET, surgical resection, or intensive surveillance. Careful consideration should be given to patient's preferences, surgical risk, and available expertise. If esophagectomy is planned, patient should be referred to a center of expertise and high volume (at least 20 esophagectomies per year) to reduce operative mortality to less than 5%. The *AGA *concludes that EET is a reasonable therapeutic option for patients with HGD, especially in those with advanced age and comorbidities. If EET is used, any visible mucosal abnormalities should be resected by EMR. With regards to question of esophagectomy, AGA states that this option should be considered in young and otherwise fit patients with HGD. The Society of Thoracic Surgeons also adopts a position very similar to the ACG and AGA for the management of BE with HGD [176].

The patients who undergo EET for dysplastic BE should continue to undergo surveillance after the therapy. The surveillance interval is dependent on the highest degree of dysplasia documented before the ablative therapy. This surveillance protocol should be continued until lack of dysplasia is documented to a reasonable degree of certainty on 3 consecutive endoscopies. However, periodic surveillance should still be continued because of the potential of BE to recur. The precise interval for these surveillance endoscopies has not been clearly defined at this time [21, 50].

## 14. Chemoprevention of Barrett's Esophagus

### 14.1. Proton Pump Inhibitors

Studies have evaluated the role of acid and acid suppression in BE. Acid exposure has been shown to induce DNA double-strand break (DSB), increase reactive oxygen species (ROS), andactivateMAP kinase pathway, suggesting its potential role in carcinogenesis [177, 178]. Observational studies have reported decreased incidence of dysplasia with chronic PPI therapy [179, 180]. In one study, 188 BE patients treated with PPI therapy for 1–13 years were prospectively followed (mean follow-up 5.1 years). During the study period, no decrease in the length of BE was noted, but 48% of the patients developed squamous islands in the BE segments. The squamous islands correlated with the duration of PPI therapy (RR: 0.43; 95% CI: 0.35–0.65) but not with the PPI dose. All 7 patients who received PPI for 12-13 years developed squamous islands [181]. Conversely, there are reports suggesting an association between chronic PPI use and increased risk of EAC. However, these findings may have been confounded by the indication for chronic PPI. Thus, the increased incidence of EAC might have been related to the original condition for which PPI was prescribed rather than the PPI itself [182]. In summary, the evidence on the chemo preventive effects of PPI for BE is equivocal and not backed by prospective studies. Nonetheless, PPI use is recommended in BE, to control the symptoms of GERD and prevent related complications like esophagitis and ulcerations. Higher than once daily dosing of PPI cannot be recommended based on available data [8].

### 14.2. Nonsteroidal Anti-Inflammatory Drugs and Aspirin

Data suggest that COX-2-derived prostaglandin E2 (PGE2) promotes tumor growth by promoting cell proliferation, migration, apoptosis, and angiogenesis. Inhibition of COX-2 attenuates cell growth and proliferation, inhibits angiogenesis, and restores apoptosis [183–185]. The cardiovascular adverse effects associated with the long-term use of selective COX-2 inhibitors have dampened the enthusiasm about their role in cancer chemoprophylaxis. However, studies have shown that chronic use of nonsteroidal anti-inflammatory drugs (NSAIDs) or aspirin can decrease the risk of colorectal cancer, gastric cancer, and several non-GI malignancies [186]. A systematic review of 9 studies including more than 1800 patients evaluated the association between aspirin or NSAIDs use and esophageal cancer. Any use of aspirin or NSAIDs was associated with a 43% reduced risk of esophageal cancer (odds ratio (OR) = 0.57; 95% confidence interval (CI) = 0.47–0.71). Frequent use of aspirin or NSAIDs was associated with 46% risk reduction (OR = 0.54; CI = 0.43–0.67), whereas intermittent use was associated with 18% risk reduction (OR = 0.82; CI = 0.67–0.99). Analyzed separately, both aspirin use (OR = 0.5; CI, 0.38–0.66) and NSAIDs use (OR = 0.75; CI = 0.54–1.0) were associated with reduced risk of esophageal cancer. The association between any use of aspirin or NSAIDs and decreased cancer risk was seen for both esophageal adenocarcinoma (OR = 0.67; CI = 0.51–0.87) and squamous cell carcinoma (OR = 0.58; CI = 0.43–0.78) [187]. Vaughan et al. prospectively followed a cohort of 350 Barrett's patients for 20,770 person months (median followup 65.5 months). Current use of NSAIDs was associated with 68% risk reduction of EAC (HR = 0.32; 95% CI = 0.14–0.76) and past use with 30% risk reduction (HR = 0.70; 95% CI = 0.31–1.58) [188]. In a recent study from Netherlands, 570 BE patients were prospectively followed for a median of 4.5 years. Use of NSAIDs (median duration 2 months) was associated with 53% lower risk of progression to HGD/EAC (*P* = 0.03) [189]. Despite the above data, there is lack of evidence to suggest that the chemopreventive benefits of NSAIDs outweigh the potential cardiovascular side effects, risk of major GI and non-GI bleeding, and nephrotoxicity associated with their chronic use. Most experts agree that it is appropriate to use low-dose aspirin in patients with BE who have a cardiovascular indication for this medication. The concomitant use of PPI, as is the case in most BE patients, should decrease the risk of serious GI complications [8].

### 14.3. Statins

Statins exert their cholesterol-lowering effect by inhibiting the enzyme 3-hydroxy-3-methylglutaryl coenzyme A reductase. However, this class of drugs has also been shown to have other pleiotropic properties. Statins promote apoptosis and inhibit proliferation in BE cells by reducing serum-stimulated Ras activity and inhibiting activation of extracellular signal-regulated kinase (ERK) and protein kinase B (Akt) [190, 191]. A case-control study of approximately 12,000 BE patients in the Department of Veterans' Affairs database showed that statin use was associated with a reduction in EAC risk (HR = 0.55; 95% confidence interval, 0.36–0.86), with a significant trend toward greater risk reduction with longer duration of statin use [192]. In the Dutch study mentioned above, long-term use of statins (median duration of 5 years) was associated with a 54% reduction in the risk of malignant progression of BE. A combination of NSAIDs and statins was associated with a risk reduction of 78% (*P* = 0.028) [189]. Although the above studies suggest an association between statin use and decreased cancer risk in BE, a cause-effect relation cannot be established based on the available evidence.

## 15. Future Directions

BE will continue to be an area of active research in the foreseeable future due to areas of uncertainty and controversy. With recent studies showing a much lower cancer risk in BE, and the growing emphasis on health care cost containment, the rationale of screening and surveillance for BE, is likely to come under greater scrutiny [193]. More research is anticipated on the development of less invasive and more cost-effective modalities for detection of BE. Further studies are needed on non-endoscopic means for BE diagnosis such as cytosponge with immunohistochemistry, spectroscopy and colorimetric techniques [194–196]. A panel of molecular biomarkers that can stratify patients based on risk of progression to cancer might help develop a more tailored approach to BE surveillance [15]. The current limitations of high-resolution white light endoscopy and protocol biopsies in detecting dysplasia call for continued research on better techniques for optical recognition of dysplasia (e.g., confocal laser endomicroscopy, spectroscopy, optical coherence tomography, chromoendoscopy, etc.) to guide targeted biopsies for a higher diagnostic yield [197–200]. Debate is likely to intensify on the appropriate management of LGD and even NDBE as the safety, effectiveness, and durability of endoscopic ablation of BE is better defined [71, 160, 174, 201]. Finally, the chemoprevention of BE is likely to remain an active area of research.

## Figures and Tables

**Table 1:** Definition of Barrett's esophagus proposed by major professional organizations of various countries [8, 9, 21, 42].

	ACG(USA)	AGA(USA)	BSG(England)	SFED(France)
Endoscopic documentation of columnar lined mucosa in the tubular esophagus required	Yes	Yes	Yes	Yes
IM required on biopsies from the tubular esophagus	Yes	Yes	No	Yes

ACG: American College of Gastroenterology; AGA: American Gastroenterological Association; BSG: British Society of Gastroenterology; SFED: French Society of Digestive Endoscopy.

**Table 2:** Incidence of neoplastic progression of low-grade dysplasia*.

Study (reference)	Number of BE patients followed	Duration of followup	Incidence of EAC/HGD
Bhat et al. [7]	8522 with BE, (no IM required)	7 years (mean)	1.4% per year (EAC)
Hvid-Jensen et al. [14]	11,028 with BE	5.2 years (median)	0.5% per year (EAC)
Sharma et al. [43]	618 with BE; LGD diagnosed in 156 during f/u	2546 patient years (mean 4.12 years)	0.6% per year (EAC)
Lim et al. [44]	357 with BE; LGD diagnosed in 34 during f/u	8 years	9/34 cases of HGD/EAC; 3.3% per year
Schouten et al. [45]	12,0852 with BE	5.7 years (median)	0.41% per year (EAC)
Curvers et al. [46]	147 LGD patients, but only 15% of these had a consensus diagnosis of LGD	51.1 months (mean)	13.4% per year in those with a consensus diagnosis of LGD

*Includes some of the important studies, not intended to include all studies published in the literature; f/u: followup; LGD: low-grade dysplasia; HGD: high-grade dysplasia; EAC: esophageal adenocarcinoma.

**Table 3:** Incidence of esophageal adenocarcinoma in high-grade dysplasia*.

Study (reference)	Number of patients with HGD	Duration of followup	Incidence of EAC
Schnell et al. [47]	75 HGD patients who didn't have EAC after 1 year f/u	7.3 years (mean)	12/75 (16%) during 7 years; 2.19% per year
Weston et al. [48]	15 patients with unifocal HGD	36.8 ± 23.2 months (mean)	4/15 (26.7%) during f/u; ~8.7% per year
Rastogi et al. [49]	236 HGD patients (meta-analysis of 4 studies)	1241 patient years	Crude = 5.57% per year; weighted = 6.58% per year

*Includes some of the important studies, not intended to include all studies published in the literature; f/u: followup; HGD: high-grade dysplasia; EAC: esophageal adenocarcinoma.

**Table 4:** Guidelines for screening for Barrett's esophagus by major professional organization [8, 9, 21, 42, 50].

	ACG (USA)	AGA (USA)	ASGE (USA)	BSG (England)	SFED (France)
Screening of general population	No	No	No	No	No
Screening of certain high-risk groups	Individualized*	Individualized*	Yes^†^	No	No

ACG: American College of Gastroenterology; AGA: American Gastroenterological Association; BSG: British Society of Gastroenterology; SFED: French Society of Digestive Endoscopy.

*After careful discussion of the potential risks, benefits, and limitations of screening with the patient.

^†^Initial screening appropriate in select patients, no further screening needed if initial EGD is normal (see text).

**Table 5:** Guidelines for Barrett's esophagus surveillance by major professional organizations in the United States.

Grade of dysplasia	ACG	AGA	ASGE^¥^
NDBE	2 EGD within 1st year, then every 3 years if still NDBE	2 EGD within 1st year, then every 3–5 years if still NDBE	2 EGD within 1st year, then every 3 years if still NDBE
LGD^†^	Repeat EGD within 6 months; if no higher-grade dysplasia, then every 1 year	Repeat EGD within 6 months; if no higher-grade dysplasia, then every 6–12 months	Repeat EGD in 6 months; if no higher-grade dysplasia, then every 1 year
HGD^†*£*^	Repeat EGD within 3 months to rule out EAC, then every 3 months	Repeat EGD within 3 months to rule out EAC, then every 3 months	Repeat EGD within 3 months to rule out EAC, then every 3 months

^†^Should be confirmed by an expert GI pathologist.

^*£*^Detailed discussion should be initiated regarding therapeutic options.

^¥^Jumbo biopsy forceps should be used to increase yield.

ACG: American College of Gastroenterology; AGA: American Gastroenterological Association; NDBE: nondysplastic Barrett's esophagus; LGD: low-grade dysplasia; HGD: high-grade dysplasia.
